# Macrophage Infiltration Initiates RIP3/MLKL-Dependent Necroptosis in Paclitaxel-Induced Neuropathic Pain

**DOI:** 10.1155/2022/1567210

**Published:** 2022-09-16

**Authors:** Dongyang Ma, Xiuli Wang, Xin Liu, Zhao Li, Jiaxin Liu, Jing Cao, Guiying Wang, Yuexian Guo, Shuang Zhao

**Affiliations:** ^1^Department of Anesthesiology, The Third Hospital of Hebei Medical University, No. 139 Ziqiang Road, Shijiazhuang 050051, China; ^2^Department of Surgery, The Third Hospital of Hebei Medical University, No. 139 Ziqiang Road, Shijiazhuang 050051, China

## Abstract

Paclitaxel (PTX) is a commonly used antitumor drug. Approximately 80% of all patients receiving PTX chemotherapy develop chemotherapy-induced peripheral neuropathy (CIPN), limiting the use of PTX. Moreover, CIPN responds poorly to conventional analgesics. Experimental evidence suggests that the neuroinflammatory response plays an essential role in paclitaxel-induced peripheral neuropathy (PIPN). Previous studies have confirmed that dorsal root ganglion (DRG) neuron necroptosis and accompanying inflammation are linked with PIPN; however, the potential upstream regulatory mechanisms remain unclear. Preclinical studies have also established that macrophage infiltration in the DRG is associated with PIPN. TNF-*α* released by activated macrophages is the primary regulatory signal of necroptosis. In this study, we established a rat model of PIPN via quartic PTX administration (accumulated dose: 8 mg/kg, i.p.). The regulatory effect of macrophage infiltration on necroptosis in PIPN was observed using a macrophage scavenging agent (clodronate disodium). The results showed that PTX increased macrophage infiltration and the levels of TNF-*α* and IL-1*β* in the DRG. PTX also upregulated the levels of necroptosis-related proteins, including receptor-interacting protein kinase (RIP3) and mixed-lineage kinase domain-like protein (MLKL) in DRG neurons and promoted MLKL phosphorylation, resulting in neuronal necrosis and hyperalgesia. In contrast, clodronate disodium effectively removed macrophages, reduced the levels of RIP3, MLKL, and pMLKL, and decreased the number of necrotic cells in the DRG of PIPN rats, alleviating the behavioral pain abnormalities. These results suggest that PTX promotes macrophage infiltration, which results in the release of TNF-*α* and IL-1*β* in the DRG and the initiation of neuronal necroptosis via the RIP3/MLKL pathway, ultimately leading to neuropathic pain.

## 1. Introduction

Paclitaxel (PTX) is a chemotherapy drug used clinically for the management of many cancers [1]. Chemotherapy-induced peripheral neuropathy (CIPN) is a major limitation associated with PTX therapy. CIPN is characterized by “glove and sock distribution” paresthesia, numbness, tingling, and spontaneous pain [2]. Approximately 80% of cancer patients receiving PTX chemotherapy experience paclitaxel-induced peripheral neuropathy (PIPN) [3, 4]. Persistent pain caused by chemotherapy reduces the patient's quality of life and often leads to reduced doses or discontinuation of life-saving treatment. Unfortunately, there is no effective treatment for CIPN currently.

While numerous studies have investigated the mechanisms of PIPN, the precise mechanism remains elusive [5]. Our recent study demonstrated that dorsal root ganglion (DRG) neurons in rats with PIPN undergo necroptosis via the RIP3/MLKL pathway, and this is characterized by increased levels of inflammatory cytokines and satellite glial cell activation [6]. Necroptosis is a new model of cell death independent of necrosis and apoptosis; it is mediated by receptor-interacting protein kinase 1/3 (RIP1/3) and mixed family kinase domain-like protein (MLKL) and has the morphological characteristics of necrotic cells together with a strong inflammatory response [7]. Although tumor necrosis factor (TNF), FAS ligand (FASL), and TNF-related apoptosis-inducing ligand (TRAIL) can all initiate necroptosis, TNF-*α* plays the primary role [8]. Increased levels of TNF-*α* have been demonstrated in PIPN rat models [9, 10]. Studies have also evaluated macrophage infiltration of the DRG in the PIPN animal model [11, 12], with activated macrophages shown to release multiple inflammatory cytokines, including TNF-*α* [13]. Monocytes are diverse, malleable blood cells that may change their phenotype in response to environmental changes, dividing into inflammatory or anti-inflammatory subsets [14]. Monocytes play an important role in immune surveillance under a steady state and can infiltrate into tissue and develop into tissue-resident macrophages that act to remove cellular debris [15]. Macrophages are identified by their expression of CD68. Clodronate disodium entrapped in liposomes is able to selectively deplete macrophages (macrophage “suicide”) [11]. Although TNF-*α* is generated and released by a variety of cell types, the increased level of TNF-*α* that is observed in injured nerves that induce chronic pain is predominantly attributed to infiltrated macrophages [16]. On the basis of these previous findings, we hypothesized that TNF-*α* released by infiltrating macrophages triggers neuronal necroptosis, eventually leading to neuropathic pain in PIPN. To test this hypothesis, this study investigated the potential initiating mechanism underlying necroptosis in PIPN. The findings of this study will inform the development of new potential strategies for the prevention and treatment of chemotherapy-related neuropathic pain.

## 2. Material and Methods

### 2.1. Materials and Reagents

The materials and reagents included PTX (TCI, JPN); clodronate disodium liposomes (FormuMax Scientific, USA); propidium iodide (PI) and Hoechst33342 (Sigma, USA); rabbit anti-RIP3, mouse anti-NeuN, and rabbit anti-CD68 antibody (Abcam, USA); rabbit anti-MLKL and anti-*β*-actin antibody (Abclonal, USA); rabbit anti-pMLKL antibody (Thermo Scientific Fisher, USA); Alexa Fluor 488 and Alexa Fluor594 (KPL, USA); a qPCR SYBR Green Master Mix kit (Thermo Scientific Fisher, USA); and TNF-*α* and IL-1*β* ELISA kits (Thermo Scientific Fisher, USA).

### 2.2. Animals and Treatments

Eight-week-old adult male Sprague-Dawley rats, each weighing 180 to 200 g, were obtained from the Experimental Animal Center of Hebei Medical University. All animals were housed at room temperature under temperature-controlled conditions and a 12-hour light/dark cycle, with free access to food and water. All experiments were approved by the local Institutional Animal Care and Use Committee and were conducted according to the guidelines of the Association for Assessment and Accreditation of Laboratory Animal Care International (AAALAC) and the Institutional Animal Care and Use Committee (IACUC). All procedures were designed to minimize animal discomfort and to reduce the number of animals needed for statistical analysis.

The animals were divided into five groups at random: blank control group (BC) (animals did not receive drug treatment and underwent pain behavior and tissue specimen testing at pre-determined time points (Supplementary data)); PTX vehicle group (PV) (animals were given an equal volume of vehicle1 (for PTX: proportional amounts of Cremophor EL and dehydrated ethanol diluted in normal saline)); PTX group (PTX) (PTX was diluted with vehicle1 as above and was given intraperitoneally at a dose of 2 mg/kg on alternate days for a total of 4 injections (0, 2, 4, and 6 days; ultimate cumulative dose: 8 mg/kg)) [11]; PTX + vehicle2 (for clodronate) group (PTX + CV) (animals were given 0.8 mL of the liposomes intraperitoneally 30 min before the first and last PTX injection); and PTX + clodronate disodium liposomes group (PTX + CLO) (0.8 mL of the clodronate disodium liposomes (containing 7 mg/mL clodronate disodium) was administered intraperitoneally 30 min before the first and last PTX injection).

### 2.3. Assessment of Pain Behavior

Pain behavioral assessments were completed on days 0, 7, and 14. The rats were given 30 minutes to adapt to the testing environment before testing commenced. During the behavioral testing, the investigators were blinded to the drug treatment setting.

Von Frey filaments were applied vertically to the plantar surfaces of right hind paw of each rat, and the filaments were bent for 10 seconds to determine each rat's mechanical withdrawal threshold (MWT). A positive reaction was defined as a quick retreat or paw flinching. Following a reaction, the next lower force was applied. In the absence of a response, the “up-down” approach was used to apply plantar stimulation that induced a 50% withdrawal reaction [17].

Thermal hyperalgesia was assessed using the plantar test (PL-200, Chengdu Taimeng Software Technology Company, CHN) based on standard methods [9]. The plantar surface of the right hind paw was exposed to a radiant heat source from beneath a glass floor. During each test session, five measures were taken. To avoid tissue injury, a 30-second cut-off period was chosen. Between subsequent testing, the hind paws were alternatively examined at 5-minute intervals. For each test, an average of five latency measures was taken.

### 2.4. In Vivo PI Staining

As previously described [18], PI (10 mg/mL) was diluted in normal saline and 10 mg/kg of PI was delivered (i.p.) to rats 1 hour before sacrifice. Frozen DRG slices were prepared and incubated for 1 hour in 0.01 M phosphate-buffered saline (PBS) with 0.3% Triton X-100. Hoechst 33342 was used to counterstain the nuclei. All sections were photographed under a confocal microscope (FV1000, Olympus) using identical settings.

### 2.5. Preparation of Frozen DRG Slices

Following induction of deep anesthesia with sevoflurane, the rats were perfused intracardially with 4% cold paraformaldehyde phosphate buffer (pH 7.4) and fixed on a board in the prone position. Longitudinal incisions were made on both sides of the spine with a scalpel, and the entire lumbar spine was dissociated and removed. The two sides of the vertebral arch plate were exposed and cut off, section by section. The lamina was removed, and the spinal cord in the spinal canal was removed. The DRG was carefully obtained from the intervertebral foramen with ophthalmic tweezers.

All obtained DRG were fixed in 4% paraformaldehyde solution at 4 °C for another 5 hours and dehydrated with gradient sucrose solution. The DRG were transferred to 30% sucrose solution after sinking to the bottom of 20% sucrose solution. The frozen DRG were continuously sliced along the sagittal plane at a thickness of 10 *μ*m using a freezing microtome (Leica, CM2000). The slices were stored at − 20 °C prior to analysis.

### 2.6. Immunofluorescence

The animals were euthanized under deep sevoflurane anesthesia and intracardially perfused with 4% cold paraformaldehyde phosphate buffer (pH 7.4). The frozen DRG slices were prepared and blocked for 1 hour in 0.01 M PBS with 0.3% Triton X-100 and 3% bovine serum albumin (BSA). Then, the sample specimens were incubated overnight at 4 °C with the primary antibodies (rabbit anti-RIP3 (1 : 200), rabbit anti-MLKL (1 : 200), mouse anti-NeuN (1 : 200), and rabbit anti-CD68 (1 : 200)) and then with the secondary antibodies (Alexa Fluor 488 and Alexa Fluor 594; 1: 500). Hoechst 33342 was used to counterstain the nuclei (1 : 5000, Sigma). All sections were photographed using an Olympus FV1000 confocal microscope under the same settings.

### 2.7. Western Blot

The DRG tissues were obtained as described above and were homogenized and then centrifuged. SDS-PAGE was used to separate the protein samples (80 *μ*g), and the samples were transferred onto polyvinylidene difluoride membranes for overnight incubation with primary antibodies (rabbit anti-RIP3 (1 : 1000), rabbit anti-MLKL (1 : 1000), rabbit anti-pMLKL (1 : 1000), and rabbit anti-*β*actin (1 : 5000)). The membranes were treated for 90 minutes at 37 °C with the appropriate secondary antibodies before being observed with Odyssey infrared imaging equipment (LI-COR, USA). The average protein level was calculated based on the tests of each sample in triplicate. The ratio of the grey value to that of *β*-actin was used to indicate the relative protein level.

### 2.8. Quantitative Real-Time PCR (qRT-PCR)

Total RNA was extracted from the DRG tissues after 14 days using TRIzol, according to the manufacturer's instructions. The RNA samples were reverse transcribed to cDNA with a cDNA synthesis kit, and then PCR amplification was performed with an LC96 qPCR real-time system (Roche, SUI) using a qPCR SYBR Green Master Mix kit. The primers were *rip3* fwd 5′-GTTTACAGTTTTGGGGTCCTCGT-3′ and rev 5′-TGGTTTTTGATTCACAGTCTTGG-3′, *mlkl* fwd 5′-TCCAGTCACCATCAAAGTATTCA-3′ and rev 5′- TCCTTGTCTCTATCCAGCAGTTC′, *gapdh* fwd 5′-ATCAAGAAGGTGGTGAAGCAGG-3′ and rev 5′-CATCAAAGGTGGAAGAATGGGAG-3′. The mRNA levels of the target genes were normalized to the mRNA levels of GAPDH using the 2^−*ΔΔ*CT^ method. All measurements were performed in triplicate.

### 2.9. Elisa

The DRG tissues were homogenized on ice. Then, the homogenate was centrifuged for 20 minutes at 12000 rpm. Proinflammatory cytokines including IL-1 and TNF-*α* were detected in the supernatant utilizing an ELISA kit, in accordance with the manufacturer's instructions.

### 2.10. Statistical Analyses

Statistical analyses were performed using SPSS 24.0. All data are presented as the mean ± SD. Comparisons were performed with one-way analysis of variance (ANOVA) followed by the least significant difference (LSD) test for post hoc analysis. *P* < 0.05 was considered statistically significant.

## 3. Results

### 3.1. Clodronate Disodium Liposomes Alleviate Abnormal Pain Behavior Induced by PTX

**Figure 1:**
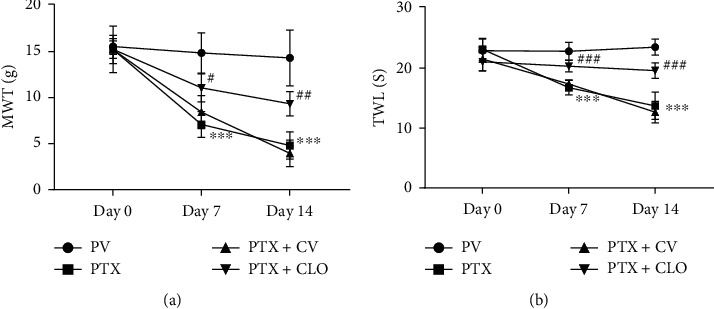
Pain behavioral assessment. (a) Effects of clodronate on mechanical withdrawal threshold (MWT) in PIPN rats. (b) Effects of clodronate on thermal withdrawal latency (TWL) in each group. Data are expressed as the mean ± SD (*n* = 6 in each group). The results were analyzed using one-way ANOVA followed by the LSD post hoc test. ^∗∗∗^*P* < 0.001 vs. VC group, ^#^*P* < 0.01, ^###^*P* < 0.001 vs. PTX + CV group. Abbreviations: PTX: paclitaxel; CLO: clodronate disodium liposomes.

To evaluate the effect of clodronate disodium on the pain behavior of PIPN rats, mechanical and thermal pain were tested (Figure 1). The pain data obtained on day zero were taken as the baseline pain behavior. There were no significant differences in MWT and TWL between the BC and PV groups at any of the time points (Supplementary Figure S1). In the PV group, there were no significant differences in MWT and TWL at any of the time points. These results indicate that the PTX solvent did not influence the experimental results. Before the experiment (day zero), there were no significant differences in MWT and TWL among the groups, indicating that the results were not significantly influenced by individual differences between the experimental animals. Compared with the PV group, MWT and TWL were significantly reduced in the PTX and PTX + CV groups, except on day zero. There were no significant differences in MWT and TWL between the PTX and PTX + CV groups. Compared with the PTX + CV group, the PTX + CLO group exhibited significant increases in MWT and TWL on days 7 and 14. These results suggest that clodronate disodium liposomes alleviate PTX-induced neuropathic pain in rats.

### 3.2. PTX Induced Macrophage Infiltration and a Neuroinflammatory Response in the DRG of Rats with PIPN, which Was Reversed by Clodronate Disodium Liposomes

**Figure 2:**
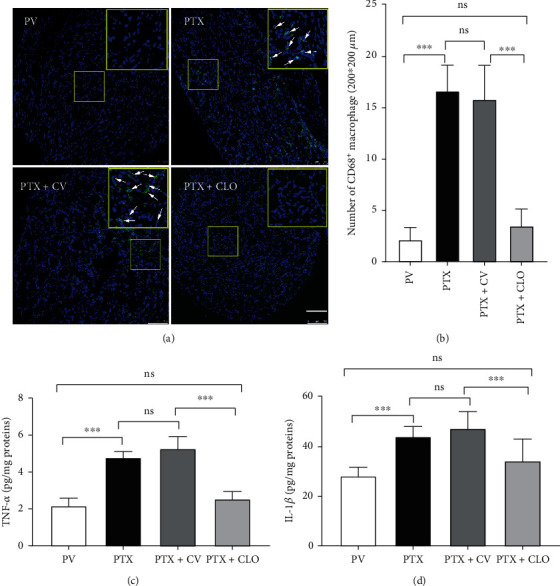
The effect of clodronate disodium liposomes on macrophage infiltration and levels of inflammatory cytokines in the DRG of rats with PIPN at day 14. (a) Microphotographs of CD68^+^ macrophages assayed by immunofluorescence in the DRG on day 14 (scale bar = 100 *μ*m). (b) Number of CD68^+^ macrophages. Three 200 *μ*m × 200 *μ*m areas were randomly selected to count the number of CD68+ cells in one slice and the average value was calculated. (c-d) Levels of inflammatory cytokines in the DRG evaluated by ELISA. Data are expressed as the mean ± SD (*n* = 6 in each group). The results were analyzed using one-way ANOVA followed by the LSD post hoc test. Asterisks indicate a significance between two groups at ^∗∗∗^*P* < 0.001; ns: no significant difference between two groups. Abbreviations: TNF-*α*: tumor necrosis factor-*α*; IL-1*β*: interleukin-1*β*; PTX, paclitaxel; CLO, clodronate disodium liposomes.

To observe the infiltration of macrophages and the inhibitory effect of clodronate disodium liposomes, macrophage infiltration and inflammatory cytokines in the DRG were observed on day 14 (Figure 2). We found almost no CD68^+^ macrophages in the DRG of rats in the PV group, while there was a significant increase in the number of CD68^+^ macrophages in the DRG of rats treated with PTX. There was no significant difference in the number of CD68^+^ macrophages between the PTX and PTX + CV groups. Compared with the PTX + CV group, the number of CD68^+^ macrophages in the DRG of the PTX + CLO group was decreased significantly (Figures 2(a) and 2(b)). These results are consistent with previous studies [11, 12], demonstrating increased DRG macrophage infiltration in rats with PIPN, which was effectively reversed by clodronate disodium liposomes. Macrophages release inflammatory cytokines that mediate the neuroinflammatory response [13]. The effect of macrophage infiltration on the neuroinflammatory response in the DRG of PIPN rats was evaluated based on the levels of TNF-*α* and IL-1*β* in the DRG (Figures 2(c) and 2(d)). Exposure to PTX significantly increased the levels of TNF-*α* and IL-1*β* in the DRG compared with the PV group. There were no significant differences between the PTX and PTX + CV groups. Compared with the PTX + CV group, clodronate disodium liposomes significantly reduced the levels of TNF-*α* and IL-1*β* in the DRG of PIPN rats. These results suggest that the elimination of macrophages in the DRG inhibits the release of TNF-*α* and IL-1*β*, thereby reducing the inflammatory response in the DRG.

### 3.3. Clodronate Disodium Inhibited the Upregulation of Necroptosis-Related Proteins in the DRG Induced by PTX

**Figure 3:**
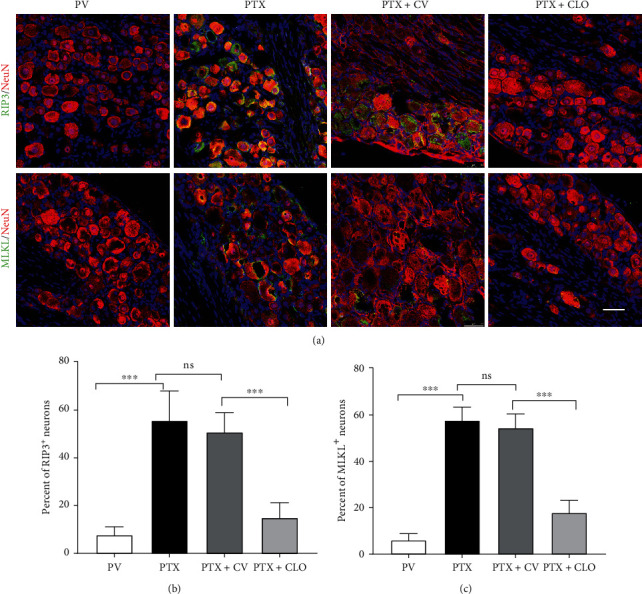
Expression of necroptosis-related proteins in DRG neurons evaluated by immunofluorescence at day 14. (a) Microphotographs show double immunofluorescence staining of RIP3/MLKL (green) and NeuN (red), scale bar = 50 *μ*m. (b) The number of neurons co-localized with RIP3/MLKL. Data are expressed as the mean ± SD (*n* = 6 in each group). The results were analyzed using one-way ANOVA followed by the LSD post hoc test. Asterisks indicate significant difference between two groups at ^∗∗∗^*P* < 0.001; ns: no significant difference. Abbreviations: RIP3, receptor-interacting protein kinase 3; MLKL, mixed-lineage kinase domain-like protein; PTX, paclitaxel; CLO, clodronate disodium liposomes.

Since DRG neurons play a key role in sensory processing, we first analyzed the expression of RIP3 and MLKL in DRG neurons via double-labeling immunofluorescence on day 14 (Figure 3). The results showed weak fluorescence intensities of RIP3 and MLKL in the DRG of the PV group, while RIP3 and MLKL in the DRG of the PTX and PTX + CV groups were mainly co-labeled with NeuN-positive cells, showing strong fluorescence. Compared with the PV group, the proportion of co-localizing cells was increased in the PTX and PTX + CV groups; however, no significant difference was found between the PTX and PTX + CV groups. Compared with the PTX + CV group, the proportion of co-localized cells was significantly decreased in the PTX + CLO group.

**Figure 4:**
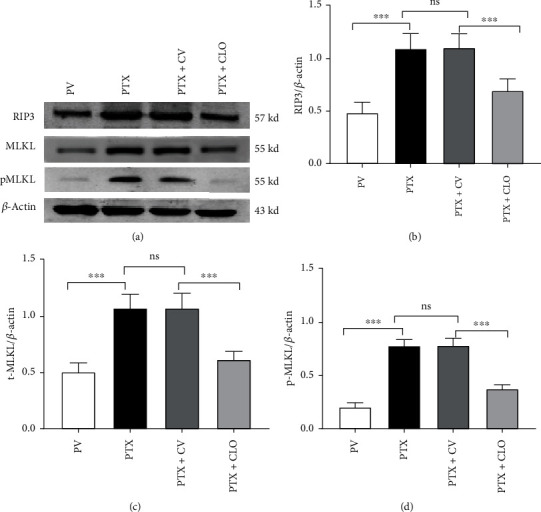
Quantitative analysis of necroptosis-related proteins in the DRG via Western blot on day 14. Imaging and quantitative analyses indicate the levels of RIP3, MLKL, and pMLKL in the DRG of rats on day 14. Data are expressed as the mean ± SD (*n* = 6 in each group). The results were analyzed using one-way ANOVA followed by the LSD post hoc test. Asterisks indicate significant difference between two groups at ^∗∗∗^*P* < 0.001; ns: no significant difference. Abbreviations: RIP3, receptor-interacting protein kinase 3; MLKL, mixed-lineage kinase domain-like protein; pMLKL: phosphorylated MLKL; PTX, paclitaxel; CLO, clodronate disodium liposomes.

**Figure 5:**
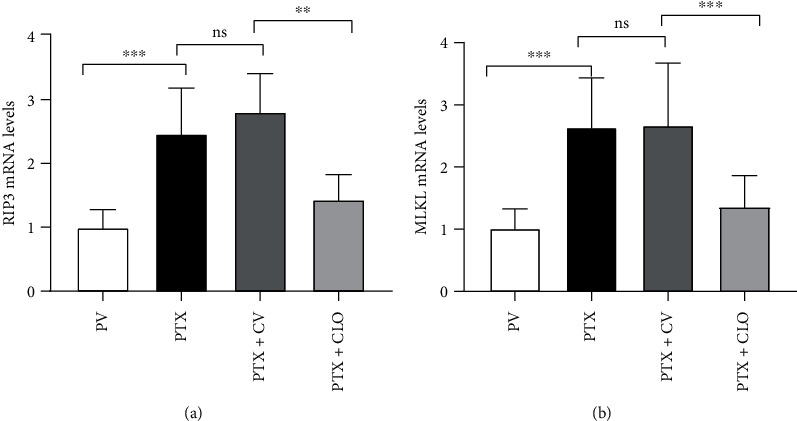
Expression of RIP3 and MLKL mRNA in rats with PIPN evaluated via qRT-PCR. Data are expressed as the mean ± SD (*n* = 6 in each group). The results were analyzed using one-way ANOVA followed by the LSD post hoc test. Asterisks indicate significant difference between two groups at ^∗∗^*P* < 0.01, ^∗∗∗^*P* < 0.001; ns: no significant difference between two groups. Abbreviations: RIP3, receptor-interacting protein kinase 3; MLKL, mixed-lineage kinase domain-like protein; PTX, paclitaxel; CLO, clodronate disodium liposomes.

Then, Western blot experiments were conducted to more accurately quantify the differences in the RIP3 and MLKL levels among the groups. We found minor levels of RIP3 and MLKL in the DRG of the PV group. Compared with the PV group, the levels of RIP3 and MLKL in the PTX group were increased significantly. There were no significant differences between the PTX group and the PTX + CV group. Compared with the PTX + CV group, the PTX + CLO group showed decreases in RIP3 and MLKL levels (Figure 4). These RT-PCR results indicate that PTX treatment increased the mRNA levels of RIP3 and MLKL, while clodronate disodium significantly inhibited these changes (Figure 5). These results suggest that PTX upregulated RIP3 and MLKL proteins in DRG neurons, while clodronate disodium significantly inhibited these changes.

### 3.4. Clodronate Disodium Liposomes Inhibited the Activation of the RIP3/MLKL Pathway Induced by PTX

Phosphorylated MLKL (p-MLKL) is regarded as the executor of necroptosis. In this study, there was no significant difference in the pMLKL level between the BC and PV groups (Supplementary Figure S2). PTX treatment increased the level of p-MLKL compared to the PV group. There was no significant difference between the PTX and PTX + CV groups. Clodronate disodium liposomes partly reversed the increase in p-MLKL induced by PTX (Figure 3), suggesting that clodronate disodium liposomes can inhibit the activation of the RIP3/MLKL pathway.

### 3.5. Clodronate Disodium Liposomes Inhibited PTX-Induced DRG Cell Necrosis

**Figure 6:**
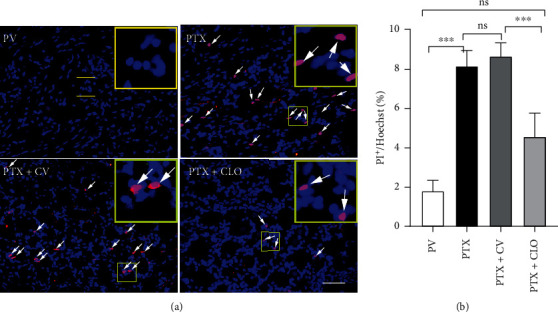
Neuronal necrosis of the DRG in rats with PIPN evaluated via PI/Hoechst staining. (a) Microphotographs of in vivo PI labeling in the DRG on day 14. (b) Quantification of PI-positive cells (scale bar = 50 *μ*m). Data are expressed as the mean ± SD (*n* = 6 in each group). The results were analyzed via one-way ANOVA followed by the LSD post hoc test. Asterisks indicate significant difference between two groups at ^∗∗∗^*P* < 0.001; ns: no significant difference. Abbreviations: PI: propidium iodide; PTX, paclitaxel; CLO, clodronate disodium liposomes.

In vivo PI staining was performed to evaluate DRG cell necrosis in experimental rats (Figure 6). Almost no PI-positive cells were detected in the DRG in the PV group. Compared with the PV group, the number of PI-positive cells in the PTX group was increased significantly. There was no significant difference in the number of PI-positive cells between the PTX and PTX + CV groups. However, macrophage clearance by clodronate disodium liposomes significantly reduced the number of positive cells in the DRG of rats with PIPN suggesting that clodronate disodium liposomes can inhibit PTX-induced cellular necrosis in the DRG.

## 4. Discussion

Patients undergoing PTX chemotherapy increasingly manifest CIPN, which is a therapeutic limitation [2, 4]. PTX can bind to *β*-tubulin to stabilize the microtubule structure, thereby inhibiting the formation of spindles and preventing cell mitosis, eventually causing mitochondrial damage and apoptosis. Most chemotherapeutic agents do not penetrate the blood-brain barrier (BBB) but can cross the blood-nerve barrier (BNB), where they accumulate in the DRG and peripheral nerves, exerting toxicity [19, 20]. In this study, we determined the role of macrophage infiltration in the pain behaviors and inflammation responses of PTX-treated rats. The rats were treated with clodronate disodium entrapped in liposomes, which is able to inhibit macrophage infiltration [11]. In the present study, clodronate disodium treatment almost completely eliminated macrophage infiltration into the DRG. Furthermore, clodronate disodium alleviated abnormal pain behaviors and reduced the elevated TNF-*α* and IL-1*β* levels in the DRG of PTX-treated rats.

Our previous study has found that RIP3/MLKL pathway-regulated DRG neuronal necroptosis is a new mechanism of PIPN [6]. Necroptosis, also known as programmed necrosis, is a cell death mechanism distinct from necrosis and apoptosis. It is primarily regulated by the RIP3/MLKL signaling pathway and is characterized by necrosis [7]. TNF-*α* is now thought to be the major signal that causes necroptosis [21] and was shown to be significantly increased in our previous study [6]. The binding of TNF-*α* to TNF-receptor-1 (TNFR1) causes trimerization and activation of the receptor. Through its own death domains (DDs), activated TNFR1 begins to form complex I and triggers RIP1/3-dependent necroptosis [22]. RIP1 and RIP3 bind to each other via their respective homotypic interaction (RHIM) domains, creating functional amyloid signaling complexes; this results in the self-phosphorylation of RIP3 at Ser227 and MLKL recruitment [22, 23]. MLKL is a RIP3 substrate that binds to RIP3 via a C-terminal kinase-like domain. Phosphorylation of MLKL eventually results in cell membrane lysis [23]. RIP3 and MLKL are often used as molecular markers to determine the degree of necroptosis [24]. In previous studies, we confirmed that PTX increases the protein levels of RIP3 and MLKL in the DRG [6]. In this study, we not only confirmed that PTX increases the mRNA levels of RIP3 and MLKL, but also found that the phosphorylation of MLKL was enhanced after PTX treatment. However, all of the above changes induced by PTX were significantly inhibited by clodronate disodium.

p-MLKL is thought to be the necroptosis executor; it binds to the cell membrane, alters the membrane permeability, and causes intracellular Ca^2+^ overload, triggering necroptosis via the altered osmotic pressure of cells [25]. The PI staining results showed that clodronate disodium significantly inhibited PTX-induced necrosis of DRG neurons. These results suggest that PTX promotes macrophage infiltration in the DRG, increasing the release of TNF-*α*, which not only upregulates the RIP3/MLKL signaling pathway but also promotes its activation, leading to DRG neuronal necroptosis and neuropathic pain. In addition, it is possible that PTX may induce DRG neuron necroptosis directly. It has been shown that PTX exerts its antitumor activity by directly triggering necroptosis in lung adenocarcinoma [26, 27] and gastric adenocarcinoma cells [28]. This process may be related to PTX-induced activation of macrophages through Toll-like receptors (TLRs) [29]. In the absence of TNF-*α*, TLR3/4 forms a complex containing the RHIM domain [30]. When caspase 8 remains inactive, MLKL is recruited and phosphorylated by RIP3, leading to necroptosis. Research indicates that TLRs signaling is involved in PTX-induced neuropathic pain [31, 32] and may represent another mechanism of necroptosis in PIPN. Thus, the role of TLRs in PTX-induced necroptosis in the PIPN process remains to be elucidated in future studies.

As for the mechanism underlying the role of necroptosis in neuropathic pain, this may be attributed to the neuroinflammatory response. Necroptosis is a pro-inflammatory death mechanism that triggers the rupture of cell membranes and the release of numerous damage-associated molecular patterns (DAMPs), such as HMGB1, TNF-*α*, and IL-1*β*, which then activates the immune cascade [33]. DAMPs play an important role in the development of neuropathic pain by activating a variety of glial and autoimmune cells [34, 35]. Not only is necroptosis a major source of inflammatory cytokines, but it is also initiated by them. Necroptosis and the neuroimmune mechanism could, therefore, constitute a positive feedback mechanism, which initiates and maintains the pain state.

In addition to DRG neuronal necroptosis caused by macrophage-derived TNF-*α*, activated macrophages may also be directly involved in the CIPN process via the release of inflammatory cytokines and chemokines. IL-1*β* is released by macrophages in response to a variety of chemotherapeutic agents such as PTX, cisplatin, vincristine, and methotrexate. IL-1*β* was one of the first cytokines to be linked to chronic peripheral pain problems because of its capacity to quickly activate nociceptive fibers. In a surgery-induced chronic pain model, IL-1*β* knockout mice showed resilience to pain [36]. TNF-*α* also rapidly and directly stimulates and sensitizes A and C fibers [37]. So there is still a long way to go to elucidate the mechanism of PIPN.

## 5. Conclusions

The current data demonstrate that paclitaxel promotes macrophage infiltration in the DRG, which initiates RIP3/MLKL pathway-dependent neuronal necroptosis. Necroptosis and the neuroinflammatory response exhibit a positive feedback loop with each other, eventually resulting in an inflammatory cascade. This novel mechanism of PTX-induced neuropathic pain may provide another possible strategy for the treatment of neuropathic pain.

## Data Availability

The study data are available from the corresponding author on reasonable request.

## Supplementary Materials

Supplementary 1 Figure S1. Pain behavioral assessment between BC and PV groups. (a) Comparison of MWT between BC and PV groups at each time point. (b) Comparison of TWL between BC and PV groups at each time point.

Supplementary 2 Figure S2. Expression levels of pMLKL in DRG between BC and PV groups via western blot on day 14.
