# Supplementary figures and images for: Macrophage Infiltration Initiates RIP3/MLKL-Dependent Necroptosis in Paclitaxel-Induced Neuropathic Pain
Source: Mediators Inflamm. 2022 Sep 16;2022:1567210. doi: 10.1155/2022/1567210 (PMC9508459; doi:10.1155/2022/1567210)

A

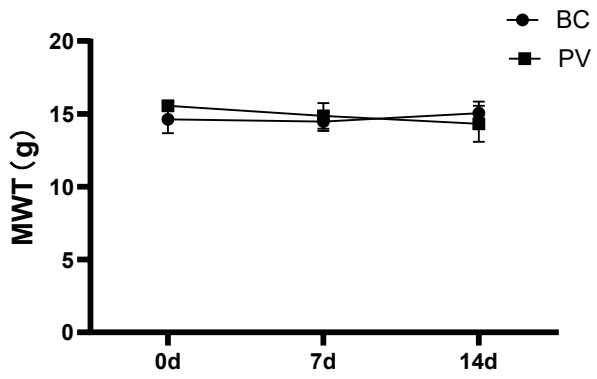

B

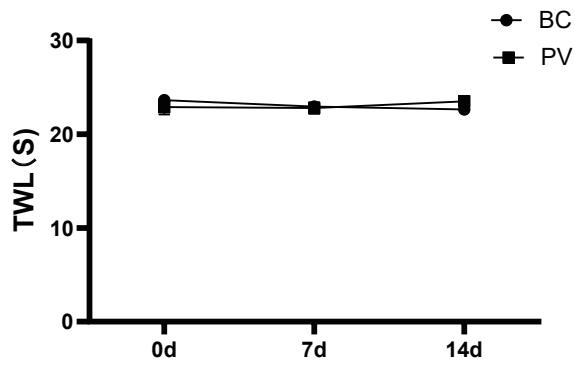

Supplement: Supplementary 1 — Figure S1. Pain behavioral assessment between BC and PV groups. (a) Comparison of MWT between BC and PV groups at each time point. (b) Comparison of TWL between BC and PV groups at each time point. [file 1567210.f1.pdf]

BC BC PV PV

pMLKL

$\beta$ -actin

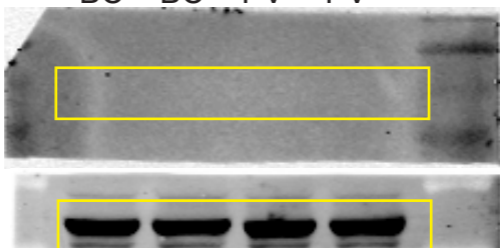

Supplement: Supplementary 2 — Figure S2. Expression levels of pMLKL in DRG between BC and PV groups via western blot on day 14. [file 1567210.f2.pdf]
